# Assessment of Allergic and Anaphylactic Reactions to mRNA COVID-19 Vaccines With Confirmatory Testing in a US Regional Health System

**DOI:** 10.1001/jamanetworkopen.2021.25524

**Published:** 2021-09-17

**Authors:** Christopher Michael Warren, Theo Thomas Snow, Alexandra S. Lee, Mihir Mukesh Shah, Anja Heider, Andra Blomkalns, Brooke Betts, Anthony S. Buzzanco, Joseph Gonzalez, R. Sharon Chinthrajah, Evan Do, Iris Chang, Diane Dunham, Grace Lee, Ruth O’Hara, Helen Park, Mohamed H. Shamji, Lisa Schilling, Sayantani B. Sindher, Deepak Sisodiya, Eric Smith, Mindy Tsai, Stephen J. Galli, Cezmi Akdis, Kari C. Nadeau

**Affiliations:** 1 Sean N. Parker Center for Allergy and Asthma Research at Stanford University, Stanford, California; 2 Center for Food Allergy and Asthma Research, Department of Preventive Medicine, Northwestern University Feinberg School of Medicine, Chicago, Illinois; 3 Swiss Institute of Allergy and Asthma Research, University of Zurich, Davos, Switzerland; 4 Department of Emergency Medicine, Stanford University School of Medicine, Palo Alto, California; 5 Stanford Health Care, Stanford, California; 6 Stanford University School of Medicine, Stanford, California; 7 Division of Pulmonary, Allergy, and Critical Care Medicine, Department of Medicine, Stanford, California; 8 Stanford Children’s Health and Stanford University School of Medicine, Department of Pediatrics, Palo Alto, California; 9 Department of Psychiatry and Behavioral Sciences, Stanford University School of Medicine, Palo Alto, California; 10 VA Palo Alto Health Care System, Palo Alto, California; 11 Immunomodulation and Tolerance Group, Allergy and Clinical Immunology, Department of National Heart and Lung Institute, Imperial College London, London, United Kingdom; 12 Centre in Allergic Mechanisms of Asthma, London, United Kingdom; 13 Department of Pathology, Stanford University School of Medicine, Stanford, California; 14 Department of Microbiology and Immunology, Stanford University School of Medicine, Stanford, California

## Abstract

**Importance:**

As of May 2021, more than 32 million cases of COVID-19 have been confirmed in the United States, resulting in more than 615 000 deaths. Anaphylactic reactions associated with the Food and Drug Administration (FDA)–authorized mRNA COVID-19 vaccines have been reported.

**Objective:**

To characterize the immunologic mechanisms underlying allergic reactions to these vaccines.

**Design, Setting, and Participants:**

This case series included 22 patients with suspected allergic reactions to mRNA COVID-19 vaccines between December 18, 2020, and January 27, 2021, at a large regional health care network. Participants were individuals who received at least 1 of the following *International Statistical Classification of Diseases and Related Health Problems, Tenth Revision *anaphylaxis codes: T78.2XXA, T80.52XA, T78.2XXD, or E949.9, with documentation of COVID-19 vaccination. Suspected allergy cases were identified and invited for follow-up allergy testing.

**Exposures:**

FDA-authorized mRNA COVID-19 vaccines.

**Main Outcomes and Measures:**

Allergic reactions were graded using standard definitions, including Brighton criteria. Skin prick testing was conducted to polyethylene glycol (PEG) and polysorbate 80 (P80). Histamine (1 mg/mL) and filtered saline (negative control) were used for internal validation. Basophil activation testing after stimulation for 30 minutes at 37 °C was also conducted. Concentrations of immunoglobulin (Ig) G and IgE antibodies to PEG were obtained to determine possible mechanisms.

**Results:**

Of 22 patients (20 [91%] women; mean [SD] age, 40.9 [10.3] years; 15 [68%] with clinical allergy history), 17 (77%) met Brighton anaphylaxis criteria. All reactions fully resolved. Of patients who underwent skin prick tests, 0 of 11 tested positive to PEG, 0 of 11 tested positive to P80, and 1 of 10 (10%) tested positive to the same brand of mRNA vaccine used to vaccinate that individual. Among these same participants, 10 of 11 (91%) had positive basophil activation test results to PEG and 11 of 11 (100%) had positive basophil activation test results to their administered mRNA vaccine. No PEG IgE was detected; instead, PEG IgG was found in tested individuals who had an allergy to the vaccine.

**Conclusions and Relevance:**

Based on this case series, women and those with a history of allergic reactions appear at have an elevated risk of mRNA vaccine allergy. Immunological testing suggests non–IgE-mediated immune responses to PEG may be responsible in most individuals.

## Key Points

**Question:**

What risk factors and mechanisms can help explain documented allergic reactions to Food and Drug Administration–authorized mRNA COVID-19 vaccines?

**Findings:**

In this case series of 22 patients with suspected vaccine allergy receiving clinical skin prick testing (SPT) and basophil activation testing (BAT) to the whole vaccine and key components (ie, polyethylene glycol [PEG] and polysorbate 80), none exhibited immunoglobulin (Ig) E–mediated allergy to components via SPT. However, most had positive BAT results to PEG, and all had positive BAT results to their administered mRNA vaccine, with no patient sample having detectable PEG IgE.

**Meaning:**

These findings suggest that non–IgE-mediated allergic reactions to PEG may be responsible for many documented cases of allergy to mRNA vaccines.

## Teaser

This case series characterizes the immunologic mechanisms underlying allergic reactions to mRNA COVID-19 vaccines.

## Introduction

As of May 21, 2021, more than 32 million cases of COVID-19 have been confirmed in the United States, resulting in more than 615 000 deaths, which have disproportionately occurred in persons aged 65 years and older. Uncontrolled transmission of the SARS CoV-2 virus continues throughout the United States and in much of the world. The reemergence of novel, more easily and quickly transmissible variants (eg, B.1.1.7; 1.351; P.1) raise concerns about further spikes in cases and a greater ensuing public health burden.

In December 2020, the US Food and Drug Administration (FDA) granted emergency use authorization to both Pfizer-BioNTech’s BNT162B2 mRNA and Moderna’s mRNA-1273 COVID-19 vaccines. Subsequent safety analyses of Vaccine Adverse Event Reporting System (VAERS) data between December 14, 2020, and January 18, 2021, estimated vaccine-related anaphylaxis events at rates of 4.7 and 2.5 cases per million doses for BNT162B2 and mRNA-1273, respectively.^1^ Of 66 confirmed anaphylaxis cases reported from 17 524 676 vaccine administrations, 95% occurred in women, and 79% and 32% of individuals with allergic reactions had a previous history of allergies and/or allergic reactions and anaphylaxis, respectively. The US Centers for Disease Control and Prevention (CDC) reviewed 3486 reports of death among individuals who had received the COVID-19 vaccinate and found “no evidence that vaccination contributed to patient deaths.”^2^

VAERS provides valuable insights into vaccine-induced anaphylaxis; however, it has limitations. Notably, VAERS is a passive reporting system requiring health care professionals to submit event reports that include vaccine lot numbers, which can be cumbersome to obtain and submit by treating clinicians. Additionally, the anaphylaxis case definition used by VAERS requires reactions to meet strict criteria, which can exclude mild reactions and some severe allergic reactions whose systemic involvement was limited by prompt treatment. Such treatment is more likely in health care workers who were overrepresented among the first wave of vaccinations, many of whom were vaccinated via occupational health programs in hospital settings. Hypervigilance toward adverse reactions to vaccines due to early publicized reports of vaccine-induced anaphylaxis and high rates of vaccine hesitancy may also lead to false-positive reports in VAERS. Given the high and growing prevalence of allergic disease in the general US population, public concern about possible vaccine-induced anaphylaxis risk among individuals with allergies, and the key role of vaccination in achieving herd immunity to COVID 19, it is essential that additional, comprehensive, and up-to-date clinical data be evaluated to further understand this important topic. Therefore, we hypothesized that life-threatening reactions to the vaccine are extremely rare and that most allergic reactions to vaccines are due to non–immunoglobulin (Ig) E–mediated pathways.^3^

As the global public health community expands vaccine access to include younger, more diverse populations who have historically exhibited higher rates of vaccine hesitancy,^4^ it is especially critical that we better understand the mechanisms underlying vaccine-induced anaphylaxis for risk stratification and improved anaphylaxis management as well as to inform further vaccine refinement. To those ends, this study provides clinical data, including skin prick tests (SPTs), basophil activation tests (BATs), and tryptase levels for a case series of vaccine-associated allergic reactions to mRNA COVID-19 vaccines from a large regional health system that was among the first in the United States to distribute these FDA-authorized vaccines.

## Methods

This case series was designed to generate hypotheses and provide proof of concept, to recognize sentinel adverse events (allergic reactions and anaphylaxis), and to study the outcomes of new treatments (novel mRNA vaccines for COVID-19). Patient data were obtained from the Stanford Research Repository, which houses all clinical data at Stanford Medicine, including the Veterans Administration Palo Alto Hospital. Study activities were approved by the Stanford University institutional review board, and all participants provided written informed consent. This study followed the reporting guideline for case series.

**Figure 1.**
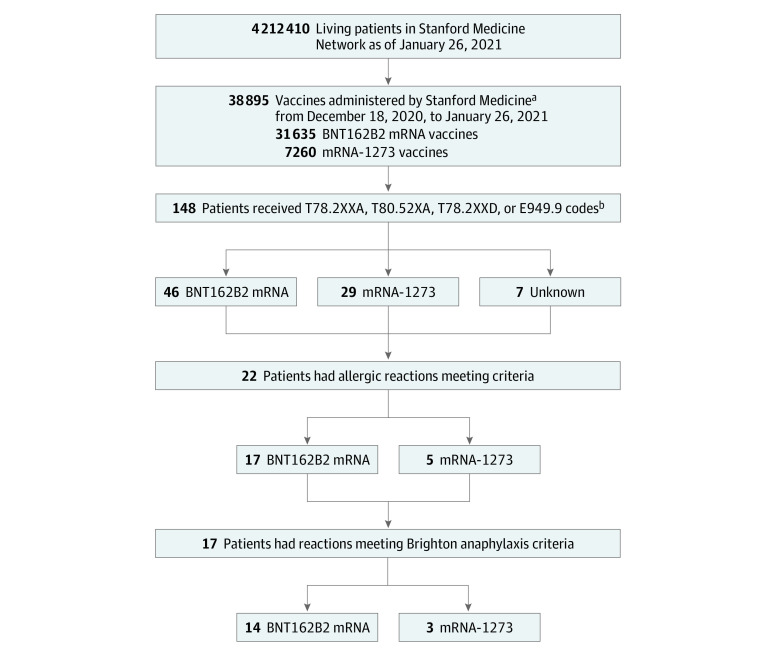
Study Flowchart ^a^Note that it is possible but not highly probable that some people received a COVID-19 dose outside Stanford Medicine during this time period. Most mRNA vaccine recipients during this time were Stanford-affiliated health care workers because public access to the vaccine was not authorized by the Santa Clara County Health Authorities for residents aged 65 years or older until January 26, 2021. ^b^T78.2XXA, anaphylaxis, initial encounter; T80.52XA, anaphylactic reaction due to vaccination, initial encounter; T78.2XXD, anaphylaxis, subsequent encounter; E949.9, vaccine or biological substance causing adverse effect in therapeutic use.

Based on multiple *International Statistical Classification of Diseases and Related Health Problems, Tenth Revision *(*ICD-10*) codes and systematic medical record review of patients with COVID-19 vaccine–associated allergic reactions, we identified those meeting prespecified criteria for suspected allergy (Figure 1). Specifically, the following search criteria were used: any patient receiving at least 1 of the following *ICD-10* anaphylaxis codes between December 18, 2020, and January 26, 2021: T78.2XXA (anaphylaxis, initial encounter), T80.52XA (anaphylactic reaction due to vaccination, initial encounter), T78.2XXD (anaphylaxis, subsequent encounter), or E949.9 (vaccine or biological substance causing adverse effect in therapeutic use). Of the 148 patients identified with 1 or more of these codes, 82 (55%) had a documented history of COVID-19 vaccination. Systematic medical record reviews of each patient identified 22 of 82 (27%) who met criteria for a possible allergic reaction. Allergic reactions were defined as those with symptoms starting within 3 hours of vaccination including hives; swelling of mouth, lips, tongue, or throat; shortness of breath, wheezing, or chest tightness; or changes in blood pressure or loss of consciousness. Reactions were graded by the authors using Brighton criteria.^5^

These individuals and their treating physicians were then contacted to invite the patient for clinical allergy follow-up testing. Each patient had been vaccinated through Stanford Medicine. Eight patients had previously received a clinical allergy workup, from which baseline tryptase levels were available. Tryptase levels were also available for these 8 patients within 2 hours after the allergic reaction and extracted from the patient’s medical record along with relevant medical history, demographic characteristics, and clinical atopic disease characteristics. Participant race and ethnicity was ascertained via medical record review and therefore, in most cases, can be assumed to result from patient self-report at clinical intake from a set of clinically defined response options. Race and ethnicity were assessed in this study to provide demographic information that may inform patient risk stratification and/or future targeted health education efforts. All participants were invited for follow-up SPT and BAT to the vaccine and relevant components, specifically polyethylene glycol (PEG) and polysorbate 80 (P80).

### SPT

Single-lancet technique was performed with DMG-PEG 2000 (Avanti Polar Lipids, 1 μg/μL) or P80 (Millipore Sigma; Sigma Aldrich, 1 μg/μL). Histamine (1 mg/mL) and filtered saline (negative control) were used for internal validation. Antihistamine medication was withheld for at least 72 hours prior to the test. Wheal and erythema were measured at 15 minutes. The wheal and erythema measurements were recorded by taking the mean of the 2 perpendicular diameters in millimeters. A wheal size of 4 mm or greater was considered positive. Saline controls were used, and all were negative. Discarded, undiluted remnant vaccine was used according to the manufacturer’s concentration instructions.

### BAT

Whole blood preserved in heparin, as described in Mukai et al,^6^ was collected from participants. Briefly, basophil activation was assessed after stimulation for 30 minutes at 37 °C with either DMG-PEG 2000 (Avanti Polar Lipids; 1 μg/μL) or P80 (Millipore Sigma–Sigma Aldrich; 1 μg/μL). Filtered saline was used as a negative control and anti-IgE (Bethyl Laboratories; 1 μg/mL) was used as a positive control.

**Figure 2.**
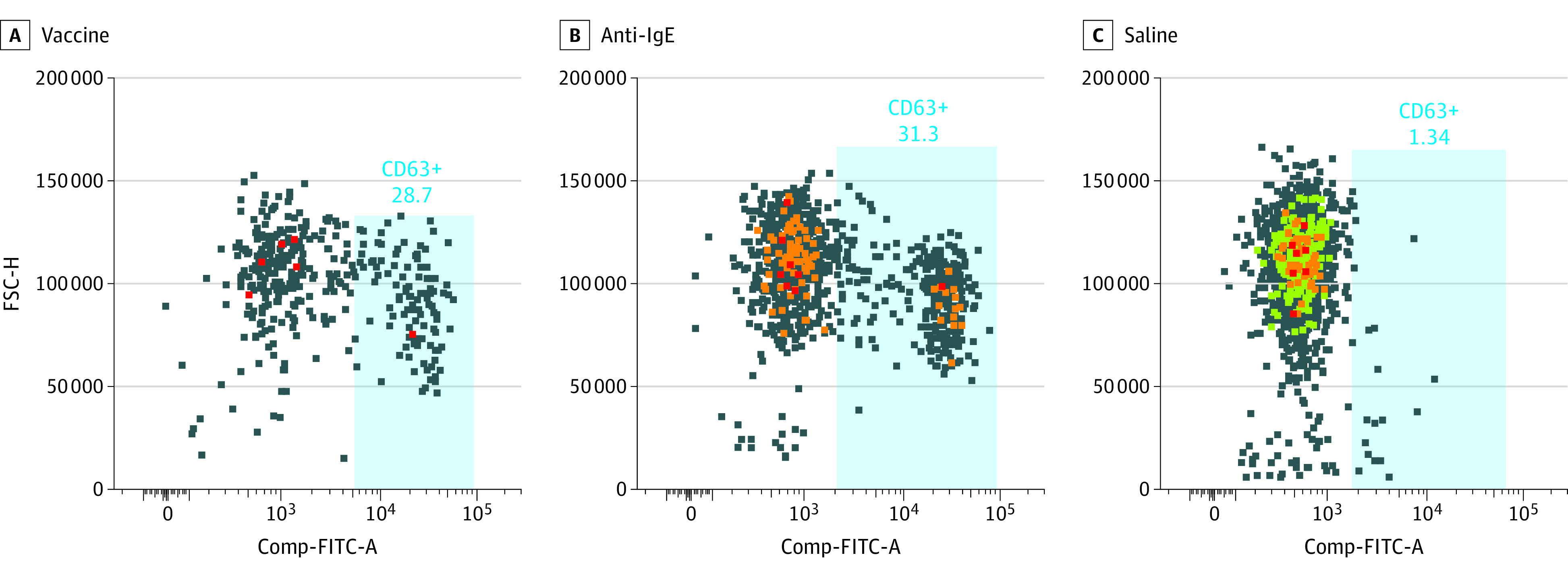
Basophil Activation Testing (BAT) Assay on Example Participant Using Vaccine, Anti–Immunoglobulin E (IgE), and Saline BAT assay on example participant with allergic reaction to the vaccine. Color indicates intensity of forward scatter and gated cells, with red being greater than orange; orange greater than green, and green greater than blue. FSC-H indicates forward side scatter-height; Comp-FITC-A, compensation–fluorescein isothiocyanate–area.

Vaccine-discarded remnant material was used at 0.007 μg/μL. All stimuli were prepared in Roswell Park Memorial Institute (RPMI) medium. Basophils were gated as CD123+HLA−DR− cells, and the percentage of CD63+ basophils was quantified by flow cytometry. Control participants were also consented using the same IRB-approved protocol, and SPT and BAT assays were performed (Table 1). Figure 2 illustrates an example of BAT results among control participants using anti-IgE (positive control), saline, and vaccine material as an activator.

**Table 1.** Characteristics of Documented Cases of 17 Systemic Allergic Reactions, 5 Allergic Reactions, and 3 Control Participants to mRNA COVID-19 Vaccines Administered at Stanford Hospital Between December 18, 2020, and January 26, 2021^a^

Age, y	Sex	Race and ethnicity	History of allergies	History of anaphylaxis	Time to onset, min	Signs and symptoms during the initial reaction	Medications received	Code type	Skin test	Brighton level	Tryptase level, ng/mL	BAT	PEG IgE levels, ng/mL	PEG IgG levels, ng/mL	Time from first dose to blood draw, d
**Documented cases of systemic allergic reactions and allergic reactions**															
20-29	F	White, non-Hispanic	No	No	10	Nausea, tongue edema	Acetaminophen, dexamethasone, epinephrine, famotidine, loratadine	Emergency	NA	2	NA	NA	NA	NA	NA
30-39^b^	F	Other, Hispanic^c^	Drug, food	Yes	1	Chest pain, fatigue, headache, heart palpitations	Diphenhydramine, epinephrine, fluticasone propionate	Outpatient	Negative to PEG, P80, and vaccine	1	NA	Positive to PEG and vaccine	<Cutoff	679.9	38
50-59	F	Black, non-Hispanic	No	No	5	Abdominal pain, dyspnea, hypotension, localized erythema, lightheadedness, presyncope, throat and chest tightness, tachycardia, airway swelling	Albuterol, diphenhydramine, epinephrine, famotidine, methylprednisolone	Emergency	Negative to PEG, P80, and vaccine	1	Baseline, 6; after reaction, 25	Positive to PEG and vaccine	<Cutoff	<Cutoff	35
40-49	F	Asian, non-Hispanic	Drug, food, latex	Drug, food	10	Cough, cyanosis, generalized pruritus, localized urticaria, tachypnea	Albuterol, dexamethasone, diphenhydramine, epinephrine, famotidine, naloxone, ondansetron, potassium chloride	Emergency	Negative to PEG, P80, and vaccine	2	Baseline, 4; after reaction, 16	Positive to PEG and vaccine	<Cutoff	349.02	76
50-59	F	Other, non-Hispanic^c^	Drug	Drug	10	Dizziness, shortness of breath, stridor	Dexamethasone, diphenhydramine, famotidine, lidocaine, magnesium sulfate, morphine, ondansetron, PEG, prednisone	Emergency	Negative to PEG, P80, and vaccine	2	Baseline, 3; after reaction, 20	Positive to PEG and vaccine	<Cutoff	805.02	44
20-29^b^	F	Native Hawaiian or other Pacific Islander, non-Hispanic	Drug, food	No	30	Generalized pruritus	Cetirizine, diphenhydramine	Outpatient	NA	Skin allergy	Baseline, 5; after reaction: 15	NA	NA	NA	NA
30-39	M	Asian, non-Hispanic	No	No	20	Generalized rash, generalized pruritus	Fluocinonide, loratadine triamcinolone acetonide	Outpatient	NA	Skin allergy	NA	NA	NA	NA	NA
30-39^b^	F	Other, non-Hispanic^c^	Drug	No	5	Dizziness, nausea, pharyngitis	Diphenhydramine, metoclopramide	Emergency	Negative to PEG, P80, and vaccine	1	NA	Positive to PEG and vaccine	<Cutoff	6903.24	14
30-39	F	White, non-Hispanic	Drug	No	150	Throat swelling, throat itching, localized angioedema; symptoms more intense following second dose; symptoms recurred at skin test	Diphenhydramine	Emergency	Negative to PEG and P80; positive to vaccine	2	Baseline, 6; after reaction, 19	Positive to PEG and vaccine	<Cutoff	1518.63	6
30-39	F	Other, non-Hispanic^c^	Drug	No	15	Generalized erythema, face edema, ocular pruritus	Acetaminophen, diphenhydramine, epinephrine, famotidine, methylprednisolone	Emergency	NA	3	NA	NA	NA	NA	NA
30-39	F	Other, Hispanic^c^	No	No	45	Diaphoresis, generalized urticaria, lightheadedness, nausea	Diphenhydramine	Emergency	NA	1	Baseline, 2; after reaction, 16	Positive to PEG and vaccine	<Cutoff	1097.98	32
30-39	F	Asian, non-Hispanic	No	No	120	Generalized erythema, generalized pruritus	Diphenhydramine, famotidine, hydrocortisone	Emergency	NA	Skin allergy	NA	NA	NA	NA	NA
30-39	F	White, non-Hispanic	Drug, food	No	15	Lightheadedness, localized erythema, localized urticaria, chest pain	Diphenhydramine, levothyroxine sodium	Emergency	NA	1	NA	NA	NA	NA	NA
30-39	F	Asian, non-Hispanic	Drug	No	1	Generalized pruritus, cough	None	Outpatient	Negative to PEG, P80, and vaccine	2	Baseline, 5; after reaction, 21	Positive to PEG and vaccine	<Cutoff	667.56	78
40-49	F	White, non-Hispanic	Food	No	15	Shortness of breath, flushed, rash, difficulty breathing	Epinephrine, prednisone	Emergency	NA	1	Baseline, 3; after reaction, 14	NA	NA	NA	NA
40-49	F	Other, non-Hispanic^c^	Environmental, food	No	120	Headache, localized urticaria, wheezing	Fexofenadine hydrochloride	Outpatient	Negative to PEG, P80, and vaccine	1	NA	Positive to PEG and vaccine	<Cutoff	491.41	0
40-49	M	Other, non-Hispanic^c^	No	No	20	Generalized erythema, generalized pruritus, generalized urticaria	NA	Emergency	NA	Skin allergy	NA	NA	NA	NA	NA
40-49^b^	F	Asian, non-Hispanic	Food	No	15	Generalized pruritus, headache, heart palpitations	Diphenhydramine	Emergency	Negative to PEG, P80, and vaccine	1	NA	Positive to PEG and vaccine	<Cutoff	2439.24	17
50-59^b^	F	White, non-Hispanic	Drug, food	Yes	5	Oral pruritus, localized erythema, throat tightness	Prednisone	Emergency	Negative to PEG, P80, and vaccine	2	NA	Positive to PEG and vaccine	<Cutoff	679.07	57
50-59	F	White, non-Hispanic	Drug	No	2	Dizziness, tachycardia, hypertension, cough	NA	Emergency	Negative to PEG, P80, and vaccine	2	NA	Negative to PEG; positive to vaccine	<Cutoff	1518.63	68
50-59	F	Other, non-Hispanic^c^	No	No	90	Hypertension, shortness of breath, palpitations	Ondansetron	Emergency	NA	2	NA	NA	NA	NA	NA
50-59	F	White, non-Hispanic	Drug, food	Drug, food	10	Oral pruritus, rash	Diphenhydramine, famotidine, methylprednisolone	Emergency	NA	Skin allergy	NA	NA	NA	NA	NA
**Control Participants**															
50-59	F	White, non-Hispanic	Drug	Drug	None	None	None	NA	Negative to PEG, P80, and vaccine	None	None	Negative to PEG and to vaccine	<Cutoff	<Cutoff	0
50-59	M	Hispanic	None	None	None	None	None	NA	Negative to PEG, P80, and vaccine	None	None	Negative to PEG and vaccine	<Cutoff	<Cutoff	31
20-29^b^	F	Hispanic	Food	Food	None	None	None	NA	Negative to PEG, P80, and vaccine	None	None	Negative to PEG and vaccine	<Cutoff	<Cutoff	67

Abbreviations: BAT, basophil activation testing; Ig, immunoglobulin; NA, not available; P80, polysorbate 80; PEG, polyethylene glycol.

^a^ Allergic reactions were defined as those symptoms that started within 3 hours of vaccination and included hives; swelling of mouth, lips, tongue, or throat; shortness of breath, wheezing, or chest tightness; or changes in blood pressure or loss of consciousness. Reactions were graded using Brighton criteria^5^ for those with systemic anaphylaxis. Tryptase was obtained at baseline in some individuals and within 2 hours for postreaction levels. Specific IgE and IgG to PEG were conducted among participants who consented for a blood draw. The blood draw was used for both the BAT and Ig assays from the same visit for the participant.

^b^ Individual received mRNA-1273 vaccine.

^c^ Races coded as other in the electronic medical record retained this categorization. No listed races or ethnicities were recoded as other for the purpose of this study.

### Anti–PEG-IgG and IgE Enzyme-Linked Immunosorbent Assays

Maxisorp 96-well microplates (NUNC) were coated with 5 μg/mL DSPE-PEG (2000) Biotin (Sigma Aldrich). After washing plates with 0.05% CHAPS (Sigma Aldrich) in PBS and blocking the wells with 2% BSA solution, the obtained plasma samples were incubated at 4 different dilutions (1:20, 1:40, 1:80, and 1:160). For the detection of specific PEG-IgG antibodies, alkaline phosphatase conjugated goat anti–human IgG (Thermo Fisher) was added at 1:2000 dilution. Specific PEG-IgE antibodies were detected by incubating samples first with a 1:3000 dilution of a mouse anti–human IgE followed by adding an alkaline phosphatase conjugated goat anti–mouse IgG (Thermo Fisher) antibody at 1:2000 dilution. After a final wash step, substrate buffer containing 1.5 mg/mL nitrophenylphosphate (NPP, Sigma Aldrich) was added, and plates were read at a wavelength of 405 nm on a microplate reader (Berthold Mithras LB940). Specific IgG and IgE antibodies to PEG concentrations of each plasma were interpolated from a standard curve created with anti-PEG human IgG and anti-PEG human IgE, respectively (Academia Sinica, Taiwan). Minimum detections cutoffs were determined as OD_405_ 0.2 and OD_405_ 0.4 for PEG IgE and PEG IgG respectively; maximum detection cutoffs were determined as OD_405_ 1.0 and OD_405_ 1.9 for PEG IgE and PEG IgG respectively. High PEG IgG was considered for levels greater than OD_405_ 1.5. The blood draw for the assays performed (both BAT and Ig levels) was done at the same visit for the each participant.

### Statistical Analysis

No statistical testing was performed. R version 4.0 (R Project for Statistical Computing) was used to generate descriptive statistics.

## Results

Between December 18, 2020, and January 26, 2021, Stanford Medicine administered 33 761 COVID-19 vaccine doses to health care workers and 5134 doses to local community members aged older than 65 years. Based on demographic information within the Stanford Research Repository, which was populated from patients’ electronic medical records, this population of vaccinated individuals was estimated to be approximately 60% women; 64% White, 2% Black, and 20% Asian; 16% younger than 50 years and 54% aged 70 years and older. These 38 895 patients are a subset of the 4 212 410 living patients present within the Stanford Research Repository during the study period. From this population, we identified 22 patients (20 [91%] women) meeting vaccine-related allergic reaction criteria (Table 1), of whom 17 (77%) received *ICD-10* anaphylaxis codes in the emergency setting, with the remainder receiving these codes in an outpatient setting. Of the 22 patients, who ranged in age from 26 to 58 years with a mean (SD) age of 40.9 (10.3) years, 15 (68%) had a physician-documented history of previous allergic reactions: 10 (45%) to antibiotics, 9 (41%) to foods (including 3 [14%] to fruit, 2 [9%] to shrimp, 1 [5%] to peanuts, and 1 [5%] to porcine products). Eight patients (36%) had a history of allergy to medications besides antibiotics, including opioids, nonsteroidal anti-inflammatory drugs, and local anesthesia (eg, lidocaine). Five patients (23%) had a history of anaphylaxis (3 [14%] to antibiotics, 1 [5%] to porcine products, and 1 [5%] to peanuts).

Of the 17 patients (77%) with mRNA vaccine-allergic reactions coded as likely anaphylaxis, each with Brighton level diagnostic certainty, 3 (14%) received epinephrine. All reactions fully resolved. Of patients who underwent SPTs, 0 of 11 tested positive to PEG; 0 of 11 tested positive to P80; and 1 of 10 (10%) tested positive to the same brand of mRNA vaccine used to vaccinate that individual. By contrast, among these same participants, 10 of 11 (91%) and 11 of 11 (100%) had positive BAT results to PEG and their administered mRNA vaccine, respectively (Figure 2). Three control participants underwent SPTs and BATs and showed typical baseline levels in control BAT assays.^6,7,8^ In Figure 2, an example BAT assay histogram is shown in which the blood of a participant who had an allergic reaction to the vaccine was incubated with vaccine, anti-IgE, and normal saline, and proportion of CD63 cells was determined (Figure 2). Table 2 reports summary findings from the BATs performed by condition and percentage of CD63+ of the gated basophil population in standardized whole blood BATs. Despite having an allergic reaction to the first, 1 patient received a second vaccine dose, which resulted in more severe symptoms. Although follow-up SPT with the same-brand vaccine material had negative results, her allergic symptoms returned with the SPT.

**Table 2.** Basophil Activation Testing With Each Condition and CD63+ of Gated Basophil Population in Standardized Whole Blood Basophil Activation Testing Assay

Overall response^a^	Experiment	CD63+ frequency of basophil, %
Negative	Anti-IgE (positive control)	38
	Saline	2
	PEG	2
	Vaccine	2
	Polysorbate 80	2
Positive	Anti-IgE (positive control)	24
	Saline	3
	PEG	4
	Vaccine	11
	Polysorbate	2
Negative	Anti-IgE (positive control)	17
	Saline	4
	PEG	4
	Vaccine	4
	Polysorbate 80	4
Positive	Anti-IgE (positive control)	31
	Saline	1
	PEG	22
	Vaccine	29
	Polysorbate 80	3
Positive	Anti-IgE (positive control)	36
	Saline	4
	PEG	22
	Vaccine	21
	Polysorbate 80	4
Positive	Anti-IgE (positive control)	38
	Saline	4
	PEG	14
	Vaccine	39
	Polysorbate 80	4
Positive	Anti-IgE (positive control)	41
	Saline	6
	PEG	73
	Vaccine	67
	Polysorbate 80	5
Positive	Anti-IgE (positive control)	11
	Saline	4
	PEG	21
	Vaccine	23
	Polysorbate 80	5
Negative	Anti-IgE (positive control)	16
	Saline	5
	PEG	4
	Vaccine	4
	Polysorbate 80	5
Positive	Anti-IgE (positive control)	15
	Saline	2
	PEG	14
	Vaccine	12
	Polysorbate 80	3
Positive	Anti-IgE (positive control)	24
	Saline	3
	PEG	25
	Vaccine	23
	Polysorbate 80	3
Positive	Anti-IgE (positive control)	23
	Saline	3
	PEG	11
	Vaccine	9
	Polysorbate 80	4
Positive	Anti-IgE (positive control)	25
	Saline	2
	PEG	17
	Vaccine	74
	Polysorbate 80	3
Positive	Anti-IgE (positive control)	74
	Saline	3
	PEG	14
	Vaccine	15
	Polysorbate 80	4
Positive	Anti-IgE (positive control)	42
	Saline	6
	PEG	61
	Vaccine	56
	Polysorbate 80	5
Positive	Anti-IgE (positive control)	77
	Saline	2
	PEG	10
	Vaccine	13
	Polysorbate 80	2

Abbreviations: Ig, immunoglobulin; PEG, polyethylene glycol.

^a^ A negative response was defined as less than 9% CD63+.

Because it is possible that the BATs were activated due to IgG (via complement activation–related pseudoallergy [CARPA]) or IgE (via IgE-FcεRec activation), we performed standard enzyme-linked immunosorbent assay to measure IgE to PEG and IgG to PEG on collected blood samples. Given that some participants had limitations with scheduling appointments for blood draws during the COVID pandemic, sampling occurred between 0 to 78 days after the first dose of the vaccine, and high levels of IgG to PEG were detected during these periods. None of the individuals with an allergic reaction had IgE to PEG greater than the cutoff value.

## Discussion

Currently, the CDC recommends that individuals with a history of allergic reaction to any mRNA COVID-19 vaccine component or who experienced a severe allergic reaction to the first dose not take either FDA-authorized mRNA vaccine.^9^ The published data to date suggest that vaccination may be specifically contraindicated among patients with allergic reactions to PEG and/or P80.^9^ The data presented here, collected from a large regional health center, suggest that allergic reactions from the mRNA vaccines are likely owing to PEG and non–IgE-mediated mechanisms, likely CARPA.

Of the stabilizing ingredients in the mRNA vaccine that we tested, P80 is a widely used emulsifier that can solubilize agents in foods and medicines, including vaccines.^10^ Previous work has found that this nonionic detergent can induce both local and systemic allergic reactions, including both IgE- and non–IgE-mediated anaphylaxis.^11^ The hydrophilic polymer known as PEG is structurally similar to P80.^12^ PEG and its derivatives are common ingredients in household products, including toothpaste, cosmetics, pharmaceuticals, and foods.^13^ In pharmaceuticals, PEG is often conjugated to biological therapeutics to form a depot agent, and sensitivity to PEG has been linked to IgE-mediated anaphylaxis after administration of PEG-conjugated biological therapeutics.^9,10,14,15,16^ Interestingly, severe allergic reactions to PEG have been associated with preexisting anti-PEG antibodies induced by PEG-containing household products,^17^ which may be more extensively used by women. Polysorbates are obtained from PEG moieties but have lower molecular weights and thus may be less allergenic.^3^ PEG may also be cross-reactive with polysorbates, which are present in some COVID-19 vaccines.^18,19^

However, measurements of preexisting anti-PEG antibodies vary widely, with a recent literature review reporting estimates ranging from 0.2% to 72% among healthy individuals.^20^ This is important because a high-molecular weight version of PEG is present in both of the FDA-authorized mRNA COVID-19 vaccines, where it helps to form a protective hydrophilic layer that sterically stabilizes the lipid nanoparticles.^21^ While further work is needed to clarify the causative role of PEG and/or P80 in the anaphylactic reactions to mRNA COVID-19 vaccines observed here and elsewhere, previous reports of similar reactions to other PEG-conjugated biologics suggest that PEG 2000 is likely to be an important causative agent that warrants further study.^22,23,24^

While allergy and/or anaphylaxis to FDA-authorized mRNA vaccines appear to be rare in all demographic groups, based on the present case series, women and those with a previous history of allergic reactions appear to have elevated risk. This is consistent with previous epidemiological data, which has found that approximately 85% of vaccine anaphylaxis cases had a history of prior allergic disease and that women are at a greater risk than men.^25,26^ Although our SPTs and BATs are research-based only, our data suggest a non–IgE-mediated immune pathway may be responsible for most reactions, possibly via complement activation through plasma immune complexes with the vaccine material or its components.^5^ This might explain the differences we observed between the SPT and whole blood BAT results, given that such PEG immune complexes likely exist in the blood more than the skin.

Future clinical trials in atopic populations—such as the ongoing National Institute of Allergy and Infectious Disease–supported phase 2 trial, Systemic Allergic Reactions to SARS-CoV-2 Vaccination (NCT04761822)—will help to elucidate mechanisms, assist with guidelines to better assess vaccine allergy risk, and inform ongoing vaccine development, such as recently announced booster shots under development to protect against COVID-19 variants. Data suggest that patients who experience allergy to mRNA vaccines, as well as those who do not experience adverse effects after vaccination, still retain relative protection against SARS-CoV-2 infection.^27,28^ Given the demonstrated safety and real-world effectiveness of these mRNA vaccines,^29^ efforts to characterize and encourage reasoned consideration of the relative risks and benefits associated with COVID-19 vaccination among patients with higher risk of vaccine allergy can also help to advance mass vaccination campaigns, including ongoing efforts to address vaccine hesitancy. For example, when considering the risks associated with COVID-19 vaccination, it is important to note that an estimated 2% to 5% of the US population have experienced anaphylaxis, most commonly to medication, food, or insect stings.^30^ However, fatal anaphylaxis is exceedingly rare, with a recent review^30^ estimating an annual incidence of fatal drug-induced anaphylaxis at lower than death due to lightning strike in the general population. In contrast, COVID-19 has killed more than 615 000 US residents and made millions ill—some for many months, with a subset who may continue to experience long-term adverse health effects.^31,32^ Moreover, allergic reactions are highly treatable, and even severe anaphylaxis usually can be promptly mitigated with appropriate preparation and medication, as all patients in the present case series experienced; each of their allergic reactions resolved.

### Limitations

This study has limitations. It is important to note that our data should not be generalized for the purposes of epidemiology of allergies to vaccines because this is a single-site study, evaluated over a limited time period, which did not incorporate a population-based sampling frame. Specific care should be taken when comparing these findings with previous reports of VAERS data^1,2^ given that the case definition used here was not intended only to identify severe allergic reactions but rather to identify cases of suspected mRNA vaccine allergy for mechanistic clinical follow-up.

## Conclusions

In this study, women and those with a previous history of allergic reactions appeared to have a higher risk of developing mRNA vaccine allergy. SPT and BAT results to whole vaccine and PEG suggest a non–IgE-mediated immune response to PEG may be responsible. In the future, testing at baseline and longitudinal measurement of IgG PEG, BATs, and other molecules will be important to further test mechanisms. If confirmed by more systematic future investigations, these findings highlight potential opportunities for patient risk stratification and for alternatives in vaccine manufacturing; furthermore, they can inform ongoing mRNA vaccine development, including that of possible COVID-19 booster shots to protect against emerging disease variants.
